# Clinical Lectures on the Physiology, Pathology and Treatment of Syphilis

**Published:** 1881-04

**Authors:** 


Clinical Lectures on the Physiology, Pathology and Treatment of Syphilis. Together with a Fasciculus of Class Room Lessons Covering the Initiatory Period. By Fessenden N. Otis, M.D., Clinical Professor of Genito-Urinary Diseases in the College of Physicians and Surgeons, New York, etc. G. P. Putnam's Sons, New York. 1881.

The reputation of the author is sufficient guarantee of the efficiency of this little volume, and needs no further commendation than the mention of the fact that the work has just been published. All who would wish to keep abreast of the times in syphilology should not delay purchasing the book.

